# Acoustic Intensity as a Potential Indicator for Congestive Heart Failure Exacerbation: An Exploratory Pilot Study

**DOI:** 10.1155/crp/3540332

**Published:** 2025-09-21

**Authors:** Eran Hadad, Jon Våbenø, Herald Reiersen, Michael Czaplik, Martin Mengel, Doron Adler

**Affiliations:** ^1^Sanolla Ltd, Nesher, Israel; ^2^Helgeland Hospital Trust, Sandnessjøen, Norway; ^3^BeneVit, Mössingen, Germany; ^4^Docs in Clouds TeleCare GmbH, Aachen, Germany; ^5^Philonmed, Heidelberg, Germany

## Abstract

**Background:** Congestive heart failure (CHF) requires continuous monitoring, especially during exacerbation phases. Pulse oximetry, commonly used for critical patient surveillance, has shown diagnostic potential in acute heart failure. In this exploratory pilot study, we investigated oxygen saturation (SpO_2_) data and respiratory sounds from CHF patients to uncover their relationship and to assess the potential of respiratory sound intensity in telehealth or self-monitoring systems, with a view toward future predictive applications.

**Methods:** The relationship between SpO_2_ and acoustic intensity was explored by collecting physiological and acoustic data, including infrasound, from 25 CHF patients using an electronic pickup device. These patients had been enrolled in a larger clinical trial that aimed to explore disease exacerbation across various chronic diseases. Four patients experienced exacerbation phases (SpO_2_ < 92%), and for each phase, we computed Pearson correlations in two frequency ranges (audible, audible + infrasound). Eight prespecified correlations were assessed, with unadjusted and adjusted *p* values (Bonferroni, FDR) and effect sizes reported.

**Results:** Significant negative correlations between specific acoustic frequency ranges and SpO_2_ variations were found in several patients. In all four patients, inclusion of the infrasound range increased the correlation magnitude compared to the audible range alone, with lower *p* values in all cases. Adjusted analyses retained significance in Patients 2 and 4 across both frequency ranges.

**Conclusion:** This pilot work identifies consistent moderate-to-strong negative correlations between acoustic intensity and SpO_2_ during CHF exacerbations. While not confirmatory, these results support the potential of acoustic intensity as a candidate indicator for early detection, warranting validation in larger studies and predictive modeling frameworks.

**Trial Registration:** EUDAMED Clinical Investigation (CIV) Identification: CIV-NO-21-10-037926

## 1. Introduction

Congestive heart failure (CHF) is a complex clinical syndrome that can result from any functional or structural cardiac disorder that impairs the ventricle's ability to fill with or eject blood [1, 2]. This can lead to a buildup of blood and fluids in the lungs and legs over time. Symptoms of CHF may include shortness of breath; fatigue; weakness; swelling in the legs, ankles, and feet; rapid or irregular heartbeat; and reduced ability to exercise [1, 2]. According to a recent study, the number of patients with heart failure worldwide nearly doubled from 33.5 million in 1990 to 64.3 million in 2017 [3]. The prevalence of CHF is increasing due to factors such as aging population, improved treatment, and survival rates in patients with ischemic heart disease, and the availability of effective evidence-based therapies prolonging life of individuals with CHF [4].

Self-monitoring or telemonitoring of CHF plays a vital role in remote data collection, enabling timely therapy adjustments and minimizing clinic visits [2, 5]. It holds promise in reducing all-cause mortality and CHF-related hospitalizations, showcasing its potential to enhance patient care and cost-efficiency when integrated with the existing healthcare services [2, 6]. However, there is room for improvement, particularly in systems aiming to anticipate and manage episodes of worsening, where the challenge of false-positive alerts persists [7].

The advent of smart stethoscopes has opened new possibilities for monitoring CHF. These devices, which are noninvasive and handheld, can collect and analyze acoustic data from the heart and lungs, thereby providing valuable insights into a patient's condition [8–10]. Several recent studies have explored innovative methods for assessing the accumulation of lung water, a critical factor in conditions such as pulmonary edema often associated with heart failure exacerbation. Yang et al. [11] have proposed an intriguing approach where acoustic-based techniques are used for lung water detection. This method offers the advantage of simplicity and portability, making it a potentially valuable tool for rapid and accurate assessment. Hong et al. [12] have conducted a detailed analysis of lung sounds in cases of excessive lung water, comparing them to healthy lung sounds. Furthermore, a pilot study by Wang et al. [13] examined respiratory sound patterns in CHF patients using acoustic-based imaging technology. They found that the intensity of acoustic lung sounds, as measured by total vibration energy, decreased significantly after the treatment of radiographically evident pulmonary edema. This finding underscores the potential utility of acoustic-based techniques not only for detecting lung water accumulation but also for monitoring the response to treatment in CHF patients with pulmonary edema. These advancements in acoustic-based methods for assessing lung water accumulation and treatment response hold promise in aiding the early detection and management of CHF exacerbation, offering potential benefits for both patients and healthcare professionals.

The infrasound spectrum, spanning low-frequency ranges, is of particular interest due to its potential to provide insights into fluid congestion within the lungs and alterations in lung compliance. Given that the presence of congested fluids in the lungs reduces lung compliance [14–16], we hypothesized that it would affect sound intensity.

Oxygen saturation (SpO_2_) by pulse oximetry has been previously examined as a diagnostic marker for acute heart failure in acute settings such as myocardial infarction [17]. It has demonstrated a value in establishing the diagnosis and severity of heart failure, and its correlation with physiological markers underscores its clinical importance. However, oxygen saturation by itself cannot predict CHF exacerbation but rather determines pulmonary disability to fully oxygenate the patient body.

Building on this premise, we present here a longitudinal pilot study that further investigates the relationship between SpO_2_ and acoustic intensity to enhance monitoring capabilities over an extended period. By capturing both physiological and acoustic data in multiple measurements taken at different time points, our findings shed light on the intricate ways that CHF patients' health and sound patterns change over time and advance the development of early exacerbation detection strategies.

Considering the limited sample size and the absence of a control group, the present work should be regarded as an exploratory, hypothesis-generating pilot study. Our objective was not to develop or validate a predictive model, but rather to examine whether consistent correlations could be detected between acoustic intensity and oxygen saturation in CHF exacerbations. This work is intended to inform the design of subsequent confirmatory studies and predictive modeling efforts.

## 2. Materials and Methods

### 2.1. Ethical Approval

Ethical clearance for this research study was granted by the Ethics Committee at the Medical Faculty of Rheinisch-Westfälische Technische Hochschule Aachen (RWTH Aachen University) under reference CTC-A-Nr: 21-222 for data collected from BeneVit, Germany, and by the Regional Committees for Medical and Health Research Ethics (REK) with reference number 252988 for data collected from Helgeland, Norway. The study in Norway was also approved by the Norwegian Medicines Agency with reference number 21/26429-14. The study was conducted in accordance with the Declaration of Helsinki and with all applicable regulations. All participants in the study provided signed informed consent, and study procedures were performed with their full understanding.

### 2.2. Data Collection

Our study specifically targeted individuals residing in nursing homes, where chronic health conditions are most prevalent. To ensure a diverse and representative sample of CHF patients from different geographic regions and healthcare systems, patients were enrolled from two different partner sites: BeneVit (Germany) and Helgeland Hospital Trust with its local nursing homes at Rana Municipality (Norway). These patients had been enrolled in a larger clinical trial that aimed to explore disease exacerbation across a number of different chronic diseases.

**Figure 1:**
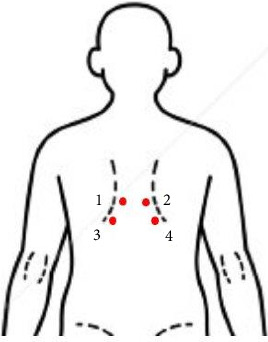
Anatomical locations of back auscultation points for lung examination.

Each participant underwent regular morning assessments over a span ranging from four to 37 weeks, depending on the patient. Within this time frame, a comprehensive set of data was collected from each individual, typically comprising four full spectrum lung sound sequences (including infrasound), oxygen saturation using pulse oximetry (SpO_2_), and additional entries detailing vital physiological parameters. The data collection process was designed to seamlessly integrate with established healthcare procedures and existing e-health and IT systems. Real-time lung sound recordings were performed in the sitting position using an electronic full spectrum pickup device, PyXy (Sanolla Ltd., Nesher, Israel). The Pick-Up unit of the PyXy consists of a microelectromechanical system (MEMS) microphone and a motion detector designed to detect mechanical vibrations (acoustic waves) from almost 0 Hz up to 100 Hz, allowing large overlapping range between these two sensors. This solution allows the measurement of very low frequencies, including the respiratory cycle which starts below 0.5 Hz. The MEMS microphone captures acoustic waves with a 16-bit analog–digital converter (ADC) and a sampling rate of 16 kHz. For each patient, four auscultation points on the posterior chest wall, middle and lower for each left and right lung fields, were sampled for at least 15 s (Figure 1).

### 2.3. Acoustic Feature Computation

The computation of acoustic features involved a series of distinct steps, each contributing to extracting valuable insights from respiratory sound signals. This comprehensive approach encompassed the detection of signal anomalies, the calculation of breathing signal envelope, and the computation of weighted sound intensity, with an emphasis on breathing intervals.

To initiate the computation, a fixed 15-s segment was extracted from each recording, starting 1 s after onset to avoid transient handling noise due to stethoscope placement and ending at 16 s. All recordings were sampled at 16 kHz.

The signal then underwent rigorous preprocessing. First, clipped samples were detected by identifying waveform amplitudes approaching the limits of the ADC's dynamic range, which may cause waveform distortion. Additionally, transient click-like artifacts were identified by computing the time derivative of the signal, extracting its envelope via the Hilbert transform, and flagging segments exceeding a predefined threshold. These two indications were combined into a per-sample binary mask, denoted as *InvalidSamples*, to improve data integrity.

Background noise was reduced using an adaptive least-mean-square (LMS) filter, with a reference signal from an auxiliary channel capturing ambient noise.

Then, the signal underwent short-time Fourier analysis (STFT [18]) using a Hann window of 512 samples (equivalent to 32 ms at a 16-kHz sampling rate) and a 256-sample hop size (50% overlap). To ensure the reliability of subsequent feature extraction, we assessed the validity of each STFT frame. A frame was considered valid if it contained no more than 20 *InvalidSamples* (from the per-sample mask) out of the 512 samples in the window, a criterion chosen to reject frames contaminated by transient artifacts (even short occurrences can disproportionately alter the energy distribution in the frame).

The subsequent step involved computing the breathing envelope, a critical metric that indicates respiration intensity. This was achieved by summing spectral amplitudes within the frequency range of 30–700 Hz for each frame, a frequency band known to encapsulate most of the respiratory signal energy [19], after excluding invalid frames. The envelope was then smoothed using a moving average filter to reduce high-frequency fluctuations while preserving the underlying respiratory dynamics.

To emphasize periods of higher respiratory activity, the breathing envelope was transformed into frame-wise weights. Envelope values between the lower and upper activity range (20th–70th percentiles) were mapped to a bounded scale, with higher weights assigned to frames corresponding to stronger breathing. After excluding invalid frames, the weighted spectrogram was averaged to obtain the respiratory sound spectrum. Respiratory intensity was then computed for two bands: the full respiratory band (30–700 Hz) and an extended low-frequency range (< 30 Hz) to capture infrasound components.

Finally, for each subject, the intensities from the four lower-back auscultation points were aggregated using a quality-weighted average. Recording quality was defined as the percentage of valid frames and converted to weights via a softmax transformation (temperature *τ* = 15), ensuring that higher-quality recordings contributed more strongly to the final aggregated values in each frequency band.

### 2.4. Statistical Analysis

We conducted correlation analyses for each of the four CHF patients who experienced exacerbation, in both the audible and audible + infrasound frequency ranges (eight comparisons in total). Given the exploratory nature and small sample size, primary results are presented with unadjusted *p* values, effect sizes (Pearson's *r*), and coefficients of determination (*R*^2^). Statistical significance for unadjusted analyses was determined using a *p* value threshold of *α* = 0.05. To assess robustness, we additionally calculated Bonferroni-adjusted *p* values (controlling the family-wise error rate) and Benjamini–Hochberg false discovery rate–adjusted *q*-values; these are reported in Supporting Table S1.

## 3. Results

Out of the total 63 patients enrolled in the study, a subgroup of 25 patients had CHF. Among these 25 CHF patients, the average age was 87 years, with a gender distribution of four males (16%) and 21 females (84%). Within this subgroup, four patients experienced CHF exacerbation phases and showed low saturation levels (below 92%) during their examinations. With a total of 72 measurements over time, these four individuals served as a unique case study within our broader dataset, offering an opportunity to investigate the dynamics of intermittent desaturation events and their implications.

**Figure 2:**
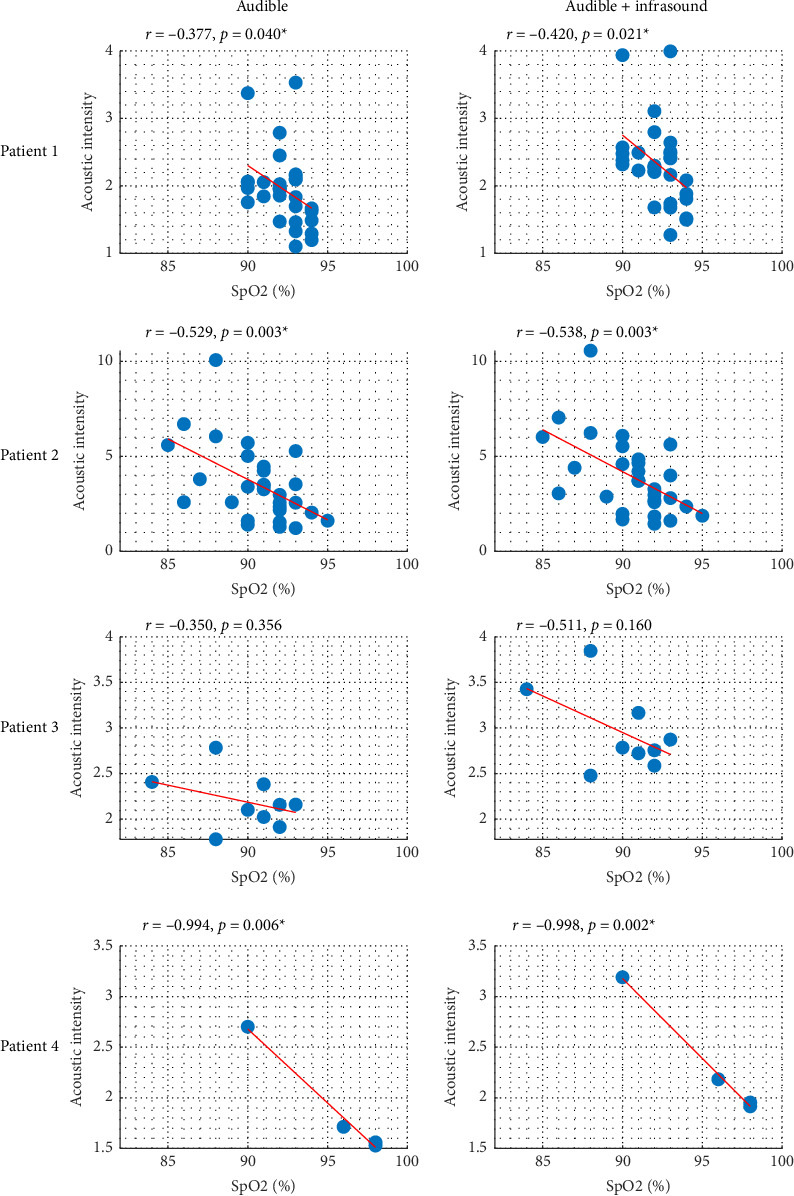
Relationship between acoustic intensity and oxygen saturation levels (SpO_2_) for each patient and within different acoustic ranges.

To establish the relationship between CHF exacerbation, measured as SpO_2_ saturation levels, and acoustic intensity, we performed a correlation analysis of the measurements taken for each of the four patients. The correlation coefficients (Table 1, Figure 2) revealed clear trends, particularly when considering the infrasound range. Thus, the infrasound spectrum appears to provide additional insights into the relationship between respiratory anomalies and saturation level variations among CHF patients. The correlations observed were consistently negative, indicating an inverse relationship between acoustic intensity and SpO_2_ levels across the patient cohort. Notably, these correlations were statistically significant for all patients except for Patient 3, whose *p* values were higher than the 0.05 threshold.

Supporting Table S1 presents unadjusted and adjusted *p* values (Bonferroni and Benjamini–Hochberg false discovery rates) for all eight predefined comparisons. After Bonferroni correction, statistically significant associations remained for both frequency ranges in Patients 2 and 4. FDR adjustment retained these and the audible + infrasound correlation in Patient 1.

## 4. Discussion

The pilot study explored the potential of acoustic intensity analysis, including infrasound frequencies, as a tool for monitoring CHF exacerbations. We observed preliminary associations between acoustic features and oxygen saturation (SpO_2_) levels in small cohort of patients, suggesting that respiratory sound patterns, particularly in the infrasound range, may hold clinical relevance. Given the small sample size, our analyses were exploratory and hypothesis-generating. No predictive modeling was undertaken, and results should not be interpreted as confirmatory evidence of predictive performance. The observed inverse relationship between acoustic intensity and SpO_2_ levels suggested that monitoring both physiological and acoustic data could provide valuable insights into the respiratory dynamics of CHF patients.

During exacerbation phases in CHF patients, as indicated by SpO_2_ readings, increased respiratory sound intensity can be attributed to several underlying mechanisms. Firstly, CHF patients often experience pulmonary edema, which is characterized by the accumulation of excess fluid in the lungs, resulting in reduced lung compliance [14, 15, 20]. This heightened stiffness and resistance to lung expansion result in increased respiratory effort and potentially louder respiratory sounds. Moreover, decreased lung compliance and increased respiratory effort may also contribute to amplified infrasound intensity, with frequencies below the threshold of human hearing emitted by the muscles and amplified as they contract [21].

Secondly, conditions such as left ventricular failure can induce airway obstruction and bronchial hyperresponsiveness [22, 23], which is characterized by excessive bronchiole contraction and narrowed airways, leading to turbulent airflow, and potentially amplifying respiratory sounds. Additionally, alterations in lung surfactant levels may play a role. Surfactant reduces alveolar surface tension, thereby aiding alveolar expansion. In cases of pulmonary edema, patients may produce surfactant-containing foamy sputum [24], contributing to narrowed airways and increased respiratory sound intensity.

Furthermore, adventitious sounds such as crackles are common in CHF and pulmonary edema [25]. Crackles, which are characterized by slight bubbling, clicking, or rattling sounds in the lungs, occur during inhalation when small airways open and close abruptly due to the presence of excess fluid or inflammation in the lung tissue. These crackles can contribute to the overall increase in sound intensity during respiratory efforts in CHF patients.

While our findings suggest that monitoring acoustic intensity can offer valuable insights into the respiratory dynamics of CHF patients, it is important to acknowledge the complexity of this physiological phenomenon, which is influenced by various factors. The relationship between lung compliance, pulmonary edema, and breathing sound intensity has been partially elucidated, but gaps in our understanding remain, necessitating further investigation. Additionally, it is noteworthy that among the four CHF patients experiencing exacerbation phases, one had only four data points, which may introduce a limitation in the depth of our analysis for this particular individual. These observations contribute to our knowledge of respiratory acoustics in pathological conditions, shedding light on the potential for future research and applications in clinical practice.

Because the present analysis involved eight predefined correlations (four patients ×  two frequency bands), the risk of Type I error from multiple testing was considered. Although our primary aim was exploratory, we performed Bonferroni and Benjamini–Hochberg adjustments for transparency (Supporting Table S1). After Bonferroni correction, statistically significant associations remained for both frequency ranges in Patients 2 and 4. FDR adjustment retained significance for these cases and for the audible + infrasound range in Patient 1. Importantly, all patients showed moderate-to-strong negative correlations (*r* = −0.35 to −0.998, *R*^2^ = 0.12 to 0.996), suggesting a consistent trend that warrants confirmation in larger studies.

In future research, a prospective longitudinal study could be employed to validate and extend our findings, involving continuous monitoring of CHF patients. A blinded approach would enhance study rigor by concealing machine learning predictions during the assessment of outcomes, such as hospitalization rates or prescription of medications indicating CHF exacerbation. This approach ensures an unbiased evaluation of the effectiveness of our proposed acoustic monitoring system. The study would specifically test a machine learning algorithm that incorporates acoustic intensity as a feature, assessing its significance and contribution to the accuracy of predicting CHF exacerbation events. The role of AI-based predictive systems is increasingly recognized in routine cardiology, where they have been applied to tasks ranging from identifying obstructive coronary artery disease using ECG waveform features during treadmill exercise tests [26] to predicting short-term mortality in acute pulmonary embolism using deep learning models [27]. Leveraging similar techniques in the context of acoustic signal analysis for CHF could enhance early detection capabilities and enable more personalized, proactive care.

## 5. Conclusion

This study illuminates a vital correlation between breathing sound intensity, especially infrasound, and CHF exacerbation phases, suggesting a valuable predictor for early detection of deteriorating health in CHF patients. The integration of physiological and acoustic data has the potential to revolutionize patient care and would be applicable across various medical conditions. By developing unique acoustic profiles during stable periods, a baseline for normal acoustic patterns can be established. Real-time monitoring and machine learning can detect subtle deviations, alerting both healthcare providers and patients. As demonstrated, it may serve as a candidate indicator for early detection of CHF exacerbations, enhancing patient engagement and care quality. Additionally, this approach holds promise for the early detection of other medical conditions, such as asthma attacks, further expanding its potential benefits.

In summary, this study lays the foundation for understanding CHF exacerbations through full spectrum breathing sound intensity correlation, having the potential to offer effective, proactive, and patient-centered care for CHF individuals.

## Figures and Tables

**Table 1:** Pearson correlation coefficients (*r*), coefficient of determination (*R*^2^), and unadjusted *p* values for the relationship between acoustic intensity and SpO_2_ in four CHF patients.

Patient	*N*	Range	*r*	*R* ^2^	*p* value
1	30	Audible	−0.377	0.142	0.040^∗^
1	30	Audible + infrasound	−0.420	0.176	0.021^∗^
2	29	Audible	−0.529	0.280	0.003^∗^
2	29	Audible + infrasound	−0.538	0.289	0.003^∗^
3	9	Audible	−0.350	0.123	0.356
3	9	Audible + infrasound	−0.511	0.261	0.160
4	4	Audible	−0.994	0.988	0.006^∗^
4	4	Audible + infrasound	−0.998	0.996	0.002^∗^

^∗^ *p* < 0.05 (unadjusted). See Supporting Table S1 for adjusted *p* values.

## Data Availability

The dataset used and analyzed during the current study are available from the corresponding author on reasonable request.

## Supporting Information

Supporting Table S1: Correlation results with adjusted and unadjusted *p* values for each patient and frequency range.
