# Supplementary material for: Acoustic Intensity as a Potential Indicator for Congestive Heart Failure Exacerbation: An Exploratory Pilot Study
Source: Cardiol Res Pract. 2025 Sep 21;2025:3540332. doi: 10.1155/crp/3540332 (PMC12476929; doi:10.1155/crp/3540332)
Supplement: Supporting Information — Additional supporting information can be found online in the Supporting Information section. [file 3540332.f1.docx]

# **Supplementary Materials**

SUPPLEMENTARY TABLE S1: Correlation results with adjusted and unadjusted p-values for each patient and frequency range.

| Patient | N | Range | r | R² | p (unadj) | p (Bonf) | q (BH FDR) |
| --- | --- | --- | --- | --- | --- | --- | --- |
| 1 | 30 | Audible | -0.377 | 0.142 | 0.040* | 0.320 | 0.053 |
| 1 | 30 | Audible+Infrasound | -0.420 | 0.176 | 0.021* | 0.168 | 0.034* |
| 2 | 29 | Audible | -0.529 | 0.280 | 0.003* | 0.024* | 0.012* |
| 2 | 29 | Audible+Infrasound | -0.538 | 0.289 | 0.003* | 0.024* | 0.008* |
| 3 | 9 | Audible | -0.350 | 0.123 | 0.356 | 1.000 | 0.356 |
| 3 | 9 | Audible+Infrasound | -0.511 | 0.261 | 0.160 | 1.000 | 0.183 |
| 4 | 4 | Audible | -0.994 | 0.988 | 0.006* | 0.048* | 0.012* |
| 4 | 4 | Audible+Infrasound | -0.998 | 0.996 | 0.002* | 0.016* | 0.016* |

* indicates p < 0.05 for the respective p-value type in that column.
